# Where does axon guidance lead us?

**DOI:** 10.12688/f1000research.10126.1

**Published:** 2017-01-25

**Authors:** Esther Stoeckli

**Affiliations:** 1 Department of Molecular Life Sciences and Neuroscience Center Zurich, University of Zurich, Zurich, Switzerland

**Keywords:** axon guidance, neural networks, neural circuit formation

## Abstract

During neural circuit formation, axons need to navigate to their target cells in a complex, constantly changing environment. Although we most likely have identified most axon guidance cues and their receptors, we still cannot explain the molecular background of pathfinding for any subpopulation of axons. We lack mechanistic insight into the regulation of interactions between guidance receptors and their ligands. Recent developments in the field of axon guidance suggest that the regulation of surface expression of guidance receptors comprises transcriptional, translational, and post-translational mechanisms, such as trafficking of vesicles with specific cargos, protein-protein interactions, and specific proteolysis of guidance receptors. Not only axon guidance molecules but also the regulatory mechanisms that control their spatial and temporal expression are involved in synaptogenesis and synaptic plasticity. Therefore, it is not surprising that genes associated with axon guidance are frequently found in genetic and genomic studies of neurodevelopmental disorders.

## It’s all about networks

Networks are an important aspect of our daily lives. But I am not talking about social networks. I am talking about the networks that make us who we are and what we do: neural networks. Neural connectivity currently is a buzz word in neuroscience. Clearly, we need to understand the formation and function of neural networks in order to explain the pathology of neurodevelopmental and neuropsychiatric disorders. Disorders such as intellectual disability, autism spectrum disorders, or schizophrenia have their roots in aberrant neural circuit formation ^1– 4^. But neural circuit formation is a multi-step process starting with cell differentiation and migration, involving axon guidance and synaptogenesis, and ending with synaptic maturation or pruning, a process that is not fundamentally different from the one ensuring synaptic plasticity in the adult nervous system. Therefore, we still struggle to understand the molecular mechanisms of neural circuit formation. In this review, I will concentrate on only one aspect of neural circuit formation: the navigation of axons to their target cells.

## Guidance molecules are conserved between different parts of the nervous system

During the last 25 years, we have learned a lot about the molecular mechanisms underlying the process of neural circuit formation. Initial studies focused on muscle innervation ^5– 7^ and wiring of the visual system ^8, 9^. Soon, dorsal commissural neurons in the spinal cord became the prime model for axon guidance studies because molecular mechanisms can best be studied in a simple system with clear readouts. Subsequently, the molecular mechanisms identified in these neurons have been tested in the establishment of more complex neural circuits in the brain ^10– 16^.

Axons that have to form long-distance connections are cutting down their pathfinding into manageable steps; that is, they use intermediate targets on their way to the final target. These intermediate targets, or choice points, are crucial for axonal navigation by providing guidance cues. Guidance cues are molecules deposited in the extracellular matrix or expressed on cells along the pathway of navigating axons. They can be subdivided into short- and long-range guidance cues. Long-range guidance cues indicate the overall direction of growth but do not specify the actual pathway. This is done by short-range guidance cues. Both types of guidance cues can be subdivided into attractive and repulsive molecules.

## Commissural axons are a useful model for axon guidance

For the dI1 subpopulation of commissural neurons in the spinal cord ^17, 18^, we know axon guidance cues and their receptors for all four categories. At the lumbar level of the spinal cord, dI1 commissural axons have a very stereotypic trajectory. Axons extend ventrally toward the floor plate, the ventral midline of the spinal cord. They cross the midline and then turn rostrally along the contralateral floor-plate border. At least some of these axons target the cerebellum ^19^. Long-range guidance cues guide them to their intermediate target, where short-range guidance cues take over to make sure that axons cross the midline before turning into the longitudinal axis. First, dI1 axons are guided ventrally by long-range repellents derived from the roof plate, the cells at the dorsal midline: BMP7 ^20– 22^ and Draxin ^23^. At the same time, axons are attracted toward the ventral midline, the floor plate, by Netrin1 binding to Dcc and Neogenin ^24– 27^. In addition, axons are attracted toward the floor plate by Shh binding to Patched and Boc ^28, 29^. Axons enter the floor plate, mediated by interactions between Contactin2 and NrCAM ^30, 31^. Based on screens in flies, the repulsive midline-associated guidance cues, the Slits and their receptors (the Robos) were then identified as the reason why axons leave the intermediate target and why they never turn back ^32– 38^. In addition to Slit, class 3 Semaphorins repel post-crossing commissural axons and prevent them from re-crossing ^39– 42^. Finally, morphogens were identified as the guidance cues directing post-crossing commissural axons toward the brain ^43– 48^.

So, case closed? Do we know everything about axonal navigation of the midline? Actually, far from it. Despite the identification of all of these molecules, we still do not fully understand how axons cross the midline and why they turn rostrally. This is not only because additional guidance cues for the navigation of the spinal cord midline were identified ^40, 49, 50^ but also because the regulation of the different receptors is not clear. Axons do not linger at the midline before they move on. Thus, the switch from attraction to repulsion has to be timed very precisely. Obviously, if axons were not attracted to the intermediate target, they would not get there; but if they did not start to be repelled upon arrival, they would not leave and move on. At the same time, they have to be equipped with receptors for the detection of guidance cues for the longitudinal axis. These receptors are not supposed to react to the guidance cues for the longitudinal axis on the ipsilateral side of the floor plate, despite the presence of the gradients of guidance molecules on both sides of the midline. So how is this precise timing of responsiveness achieved? Are similar mechanisms responsible for the formation of more complex circuits in the brain? Why so many cues for a seemingly rather simple decision at a choice point?

## Do we need more axon guidance cues to understand midline crossing?

In the beginning of the molecular era of axon guidance, the focus was on the discovery of new families of axon guidance cues and their receptors ^51– 53^. This has clearly changed. In recent years, the discovery of new guidance cues has been a rare event, but it has still happened. For instance, lyso-phosphatidyl-Β-D-glucoside, a glycerophospholipid, has been identified as a subpopulation-specific guidance cue for sensory afferents in the dorsal spinal cord ^54^. PRG2/Lppr3 (plasticity-regulated gene-2), a molecule interacting with lysophosphatic acid, was shown to regulate guidance of thalamocortical axons ^55^. But clearly, the focus in axon guidance research today is on the characterization of regulatory mechanisms. Axons are not guided by static interactions between guidance cues and their receptors on the growth cone. Rather, the responses of axons to their environment are induced by dynamic changes of these interactions. At the level of guidance cues, changes can lead to the synergistic activation of signaling pathways, as shown, for instance, for Netrin and Ephrin signaling during hindlimb innervation ^56^. Pre-crossing axons are attracted to the floor plate by a co-operation between Shh and Netrin ^57^.

The interaction between receptors can result in silencing or enhancement of a response. For example, in contrast to commissural axons, which are attracted by floor-plate-derived Netrin1, motoneurons are repelled by Netrin1 ^58^. Netrin1 is attractive for axons expressing Dcc but repulsive for axons expressing both Dcc and Unc5. Furthermore, the interaction between Dcc and Robo1 in commissural axons was shown to silence the attractive response of Netrin on post- compared with pre-crossing axons ^59^. More recently, many more of these receptor interactions in the plane of the growth cone membrane have been identified as important regulators of growth cone behavior (see “Some guidance receptors are regulated at the protein level” section).

## Axons at choice points switch their responsiveness in a precisely timed fashion

**Figure 1.**
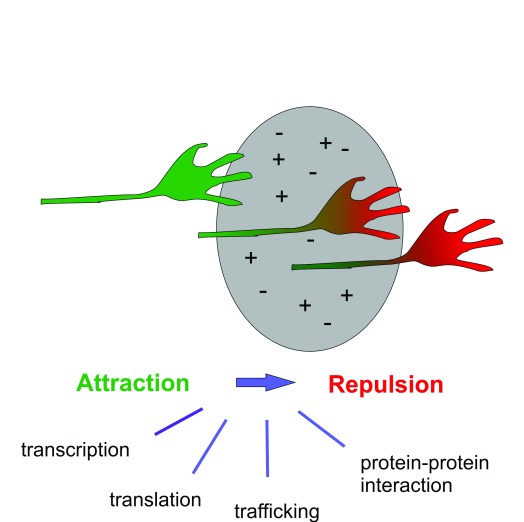
Growth cones change their responsiveness to the intermediate target upon arrival. The intermediate target is attractive for axons before contact. However, upon contact with the intermediate target, the growth cone changes its surface receptors. The expression of new receptors allows for the perception of previously undetectable guidance cues associated with the intermediate target. The alterations in surface expression of guidance receptors can be due to changes in transcription, translation, trafficking, or clustering of receptors in the growth cone membrane.

Another problem that has become, and still is, a focus of axon guidance research is the aspect of timing. Precise timing of the switch from an attractive to a repulsive behavior is, of course, required at any choice point, but again it is best illustrated with the example of midline crossing. Clearly, the switch in axonal behavior is caused by a change in the expression of guidance receptors on the growth cone surface. This is a seemingly simple change, but the abundance of possibilities that is actually found is surprising ( Figure 1). Theoretically, expression of guidance receptors on the growth cone surface can be modified by changes in gene transcription, translation, or protein transport as well as protein stability. In fact, all of these possibilities have been confirmed experimentally to occur in axons crossing the midline either in the spinal cord or in the visual system.

### Some guidance receptors are regulated at the transcriptional level

Transcriptional changes have been demonstrated to account for the switch in responsiveness to Shh, an attractive guidance cue for pre-crossing commissural axons ^28^ but repulsive for post-crossing axons ^44, 60^. This switch in responsiveness is due to the expression of Hhip (Hedgehog-interaction protein) on post- but not pre-crossing axons. Interestingly, the transcription of Hhip is triggered by Shh itself in a Glypican1-dependent manner ^60^.

### Some guidance receptors are regulated at the RNA level

Translation was shown to regulate the responsiveness of retinal ganglion cell axons to repulsive class 3 Semaphorins in the visual system of frogs ^61^. Class 3 Semaphorins bind to receptor complexes formed by PlexinAs and Neuropilins ^62^. The expression of Neuropilin1 was found to be regulated by microRNAs which bind to the 3′ untranslated region of target mRNAs and regulate their translation. In retinal ganglion cells of *Xenopus* tadpoles, mi125 prevents the expression of Neuropilin1 and therefore regulates the onset of axonal sensitivity to Semaphorin3A ^61^.

Regulation at the RNA level has also been postulated in a mechanistically distinct manner. Local translation of mRNA in the growth cone could influence axon guidance behavior, although it is not yet clear how timing is controlled ^63^. RNA-binding protein IMP2 is required for commissural axon guidance in the spinal cord. IMP2 is enriched in axons and could be responsible for local translation of axon guidance cues ^64^. Furthermore, the RNA-binding protein Nova2 was implicated in Dcc regulation in the spinal cord ^65^ and in the regulation of several axon guidance molecules in the cortex ^66^.

Stability of mRNA can be used as a regulatory means. Colak and colleagues described nonsense-mediated decay as a mechanism to modulate the stability of mRNA and thus to influence the level of protein synthesis ^67^. A direct effect on protein stability was also demonstrated as a means to regulate axonal responsiveness (see the following section). ****

### Some guidance receptors are regulated at the protein level

Axonal sensitivity to axon guidance cues can be regulated by ectodomain shedding. In particular, ADAM family members (also known as α-secretases) have been implicated in the cleavage of axon guidance receptors. In turn, these proteases are regulated by a variety of interactions with axon guidance molecules, such as Secreted frizzled-related proteins (Sfrps) ^68^. The complexity of these regulatory mechanisms was demonstrated in a recent study from the Pasterkamp lab ^69^. Lrig2 was shown to regulate axonal sensitivity to RGMa, a repulsive axon guidance cue. This was achieved by ADAM17-mediated cleavage of Neogenin, the RGMa receptor, whereas ADAM17 activity was regulated by Lrig2.

Another receptor shown to be regulated by proteolytic activity is PlexinA1, a component of the receptors for repulsive class 3 Semaphorins. On pre-crossing axons, levels of PlexinA1 are low because PlexinA1 is cleaved by Calpain1 ^40^. Upon midline contact, Calpain is inactivated by floor-plate-derived NrCAM and Gdnf, resulting in the expression of PlexinA1 on post-crossing commissural axons. The stability of PlexinA1 is regulated also by the cooperation of GDNF and NCAM ^40, 42^.

Regulated proteolytic cleavage is not restricted to receptors. Proteolytic cleavage of ligands has been demonstrated to affect midline crossing of commissural axons. Slit has long been known to be cleaved into an N- and a C-terminal fragment ^70^. However, only recently, it has been shown that both fragments affect midline crossing albeit by distinct receptors ^71^. PlexinA1, a component of the receptor for class 3 Semaphorins, was found to bind to the C-terminal part of Slit. The well-known receptors for Slit are the Robos ^37, 38^. Thus, Slit repulsion is mediated not only by Robo receptors (via the N-terminal part of Slit) but also by PlexinA1 ^71^.

The sensitivity of axons to Slit not only is determined by the expression of Robo1 but is regulated by the expression of Robo3 ^37, 72^, a divergent Robo family member ^73^. In contrast to Robo1 and Robo2, mammalian Robo3 does not bind Slit but rather regulates attractive responses to Netrin by binding to Dcc ^73^. This mechanism extends or replaces the model of a previous study, in which the expression of Robo3.1 was suggested to prevent premature sensitivity of Robo1 to midline-derived Slit ^72^. According to this study, Robo3.1, the splice isoform of Robo3 expressed on pre-crossing axons, would interact with Robo1 in cis (that is, in the plane of the growth cone membrane) and thus prevent its interaction with Slit. Post-crossing axons would express a different isoform of Robo3, Robo3.2, which would not be capable of cis-interaction with Robo1.

Finally, an additional level of regulation at the protein level that is experimentally very difficult to study is the role of direct protein-protein interaction or clustering on the growth cone surface. Initial observations that protein-protein interactions can determine the responsiveness of growth cones to individual guidance molecules were made many years ago. More recently, many more of these cis-interactions were identified: Semaphorin6B interacting with PlexinA2 in cis on pre-crossing commissural axons was shown to prevent premature sensitivity to floor-plate-derived class 3 Semaphorins ^50^. In this case, it was hypothesized that the availability of PlexinA2 for a response to midline-associated class 3 Semaphorins would be restored by the competition between Semaphorin6B/PlexinA2 cis-interactions versus trans-interactions between growth cone Semaphorin6B and floor-plate PlexinA2. Thus, although the details differ, both PlexinA1 ( 40, see above in this section) and PlexinA2 ^50^ appear to be regulated at the protein level.

Similarly, protein-protein interactions as a regulatory means for midline crossing have been reported for the visual system ^74^. Contralaterally projecting retinal ganglion axons depend on complexes between NrCAM, PlexinA1, and Semaphorin6D for midline crossing. Semaphorin6D alone is repulsive, but in combination with PlexinA1 and NrCAM, the repulsion is converted to a growth-promoting effect.

A regulatory effect of specific protein-protein interactions in the plane of the membrane (cis-interactions) on the selection of trans-binding partners has been found for sensory afferents. When they enter the dorsal spinal cord in the dorsal root entry zone, axons sort depending on their subtype. The formation of homo- versus hetero- philic cis-interactions between SynCAMs has been demonstrated to regulate their choice of trans-binding partners and thus affects sorting and pathfinding of sensory afferents in the spinal cord which express a specific set of SynCAMs ^75^.

In the formation of thalamocortical connections, Flrt3 has been identified as a co-receptor for Robo1 that can modulate axonal responsiveness to Netrin ^76^. Robo1/Flrt3 cis-interactions resulted in the upregulation of Dcc on rostral thalamocortical in contrast to intermediate thalamocortical axons. The observed upregulation of Dcc, the receptor that mediates attraction to Netrin1, could explain why rostral thalamocortical axons were attracted to an area of their intermediate target, the corridor cells, where Slit was expressed. Thus, different populations of thalamocortical axons responded distinctly to the molecular guidance cues found in the environment: Netrin and Slit. Slit binding to Robo1/Flrt3 receptor complexes primed axons to be sensitive to Dcc, explaining the previously recognized requirement of both Netrin and Slit for correct pathfinding of thalamocortical axons ^77, 78^.

Similar ‘co-operations’ of guidance cues have been identified in studies looking at hindlimb innervation ^56^. Motor axons integrate signals derived from Netrin and Ephrin. Careful quantification of the effects of loss of function of one or both guidance cues allowed for the distinction between additive and synergistic effects of these guidance cues. For a detailed explanation of the difference between the additive and synergistic interactions of guidance cues, the reader is referred to a recent review ^79^.

## Selective trafficking of guidance receptors can regulate axonal responsiveness at choice points

Endo- and exo- cytosis of guidance receptors have been demonstrated to be required for a growth cone’s response to repulsive or attractive guidance cues, respectively ^80– 83^. However, beyond these mechanistic aspects, specific changes in trafficking of guidance receptors have been shown to be involved in the temporal control of receptor expression ^84^. First demonstrated in flies, the expression of Robo on the growth cone surface was demonstrated to be dependent on specific trafficking in a Commissureless-dependent ^85– 87^ but Rab-independent ^88^ manner. In vertebrates, a Commissureless ortholog is not found, but trafficking as a means to control surface expression of Robo is nonetheless maintained in vertebrates ^38, 89^. In fact, the possibility to transport guidance receptors to the growth cone surface by shuttling them wrapped in subpopulations of vesicles not only provides specificity but also allows for an efficient change of the repertoire of guidance receptors, as large numbers of molecules can be inserted upon a stimulus that triggers the fusion of a specific subset of vesicles. A recent study has demonstrated the presence of Robo1 and Frizzled3 guidance receptors in different subpopulations of vesicles that were trafficked in a Calsyntenin1-dependent manner ^89^. Calsyntenin1 acts as a linker between the vesicles with specific cargo and the kinesin motor. RabGDI appears to be part of the stimulus that regulates the timing of vesicle trafficking, but only for those vesicles carrying Robo1 as cargo. The vesicles containing Frizzled3, the receptor for Wnt signaling regulating antero-posterior guidance of post-crossing commissural axons, also depend on Calsyntenin1 for transport, but RabGDI is not required for the delivery of Frizzled3 to the growth cone surface.

## Where do we go?

Although we may know most axon guidance molecules today, we still need to learn much more about axon guidance mechanisms before we can explain how neural circuits form. This is even true for simple circuits, such as those formed by spinal cord interneurons! In particular, we will need to understand how the interactions of guidance receptors with their ligands trigger specific intracellular signals and how they are translated into changes in growth cone behavior. While we start to understand individual signaling pathways, we do not have a clear idea how they interfere with each other. Furthermore, temporal changes in signaling are poorly understood. We have identified roles for individual molecules during different developmental processes contributing to the formation of neural circuits ^1– 3^, but it is still unclear how binding partners or the downstream signaling (or both) change over time. So we will need not only to test molecules and mechanisms identified in simple circuits in complex brain circuits but also to conceptually and experimentally integrate different signaling pathways to understand the behavior of growth cones and axons during neural circuit formation. The analysis of single-gene knockouts is not sufficient to understand the dynamic role of a protein during neural circuit formation. Eventually, we will have to study neural circuit formation at the protein level. This remains a challenge because tools are not yet available to visualize protein function *in vivo*. We can follow molecules in isolated cells *in vitro* with sophisticated high-resolution imaging methods. We can also image neuronal activity *in vivo* in actively behaving animals. But there is a huge gap in between that needs to be closed before we understand formation and function of neural circuits. However, the development of new tools and the refinement of existing technology will allow us to tackle some of these open questions in a variety of model systems and thus to assemble more and more pieces of the puzzle.
